# Psychosocial Impact of Dental Esthetics in Adolescents With Malocclusion: A Patient‐Reported Study

**DOI:** 10.1111/jerd.70237

**Published:** 2026-07-24

**Authors:** Matea Jurgec, Sara Pavlović, Barbara Živaljić, Marija Čandrlić, Stjepan Špalj

**Affiliations:** ^1^ Health Centre of Varaždin County Varaždin Croatia; ^2^ Health Centre of Karlovac County Karlovac Croatia; ^3^ Health Centre of Osijek‐Baranja County Osijek Croatia; ^4^ Faculty of Dental Medicine and Health Osijek Josip Juraj Strossmayer University of Osijek Osijek Croatia; ^5^ Faculty of Dental Medicine University of Rijeka Rijeka Croatia

**Keywords:** adolescents, dental esthetics, malocclusion, psychometrics, reliability, responsiveness, validation

## Abstract

**Introduction:**

Malocclusion and altered dentofacial esthetics significantly influence adolescents' psychosocial well‐being during a sensitive developmental period. Understanding these impacts is important for esthetic and orthodontic treatment planning.

**Methods:**

A total of 254 adolescents from orthodontic clinics in Osijek, Rijeka, and Split completed the Malocclusion Impact Questionnaire (MIQ), the Psychosocial Impact of Dental Esthetics Questionnaire (PIDAQ), the Jaw Functional Limitation Scale (JFLS), along with Likert‐type scales assessing satisfaction with dental and facial appearance and perceived need for orthodontic treatment.

**Results:**

The Croatian MIQ showed a unidimensional structure with excellent internal consistency (*α* = 0.92). MIQ correlated strongly with PIDAQ domains (*r* = −0.58–0.76, *p* < 0.001) and the esthetic component of the IOTN as rated by both patients and orthodontists (*r* = 0.27 and 0.32; *p* < 0.001), but not with JFLS or objective malocclusion indices. Malocclusion impacts were influenced by the increased esthetic component of IOTN (6% of variance; *p* < 0.001), female gender (13%; *p* < 0.001), and increased age (3%; *p* = 0.011).

**Conclusion:**

Older female adolescents experienced a greater psychosocial impact of malocclusion. The Croatian MIQ is a valid and reliable instrument for assessing the psychosocial impact of malocclusion.

## 1 Introduction

Malocclusions and altered dentofacial esthetics influence an individual's psychological well‐being, self‐esteem, and social interactions, even when oral function remains uncompromised [1, 2]. In adolescence, when self‐image and peer acceptance are particularly sensitive, dental appearance often plays a crucial role in quality of life and social confidence [3, 4]. Therefore, oral health–related quality of life (OHRQoL) instruments provide an important insight into the psychosocial consequences of malocclusion and the benefits of orthodontic treatment [5].

Growing evidence indicates that the impact of malocclusion on OHRQoL is primarily mediated through dental esthetics and smile perception rather than functional impairment. Previous studies have shown that the relationship between malocclusion and quality of life cannot be fully explained by normative clinical measures alone. Adolescents who completed orthodontic treatment reported fewer oral health‐related impacts than untreated individuals, and the combination of clinical treatment‐need indices with patient‐reported outcome measures provided a better assessment of perceived appearance and treatment benefits than clinical indices alone [6, 7]. Furthermore, psychosocial impacts of dental esthetics have been associated with female gender, self‐perceived treatment need, and the severity of anterior irregularities, which supports the importance of patient‐centered assessment of malocclusion [8, 9]. A recent meta‐analysis reported moderate‐quality evidence that malocclusion increases the risk of impaired OHRQoL in adolescents, with esthetic disturbances being the primary contributors to reduced well‐being [10]. In addition, a systematic review concluded that orthodontic treatment is associated with improvements in oral health‐related quality of life among children and adolescents [11]. These findings emphasize the need for condition‐specific patient‐reported outcome measures that are sensitive to esthetic‐ and appearance‐related concerns, which are not fully captured by generic OHRQoL instruments.

Several OHRQoL instruments have already been translated and validated in the Croatian context, including the Psychosocial Impact of Dental Esthetics Questionnaire (PIDAQ) [12], the Child Perceptions Questionnaire (CPQ11–14) [13], the Jaw Functional Limitation Scale (JFLS) [14], and the Oral Health Impact Profile (OHIP‐14 CRO) [15], all demonstrating good psychometric properties and cultural applicability. However, these instruments are primarily generic and do not specifically capture the psychosocial impact of malocclusion in adolescents. This limitation highlights the need for condition‐specific patient‐reported outcome measures that are sensitive to malocclusion‐related esthetic and psychosocial concerns.

To address this limitation, Benson and colleagues developed the Malocclusion Impact Questionnaire (MIQ) in the United Kingdom as a condition‐specific instrument designed for individuals aged 10–16 years [16]. The MIQ evaluates the psychosocial and functional consequences of malocclusion through three dimensions—psychological, social, and functional impact. Since its introduction, the MIQ has demonstrated strong psychometric performance in multiple cross‐cultural validations. Studies in Serbian and Moroccan populations confirmed high internal consistency and construct validity in adolescents seeking orthodontic treatment [17, 18]. Spanish and Chinese adaptations further supported the unidimensional structure of the instrument, while also demonstrating cultural differences in the relative contribution of psychosocial domains [19, 20]. In contrast, more recent validations in New Zealand, Finnish, and Polish samples confirmed the overall reliability and validity of the instrument, with the Finnish and Polish studies additionally reporting item‐level variability and factor‐structure modifications related to cultural context and the application of advanced psychometric methods [21, 22, 23].

However, to date, no validated Croatian version has been available, limiting cross‐cultural comparisons and the assessment of orthodontic treatment outcomes in Croatian adolescents. Therefore, the aim of this study was to translate and cross‐culturally validate the MIQ in the Croatian cultural context, and to analyze the structure of malocclusion impacts and influencing factors. The following research hypotheses were defined:

*The Croatian version of the MIQ demonstrates good psychometric properties (validity and reliability)*.

*MIQ scores are primarily associated with psychosocial and esthetic factors rather than clinical indices of malocclusion*.

*The Croatian MIQ is sensitive to changes following orthodontic treatment*.

## 2 Materials

Written informed consent was obtained from parents or guardians, with verbal assent from all adolescent participants. The research was conducted in accordance with the Declaration of Helsinki.

The English version of the MIQ was translated into Croatian following a standard forward‐backward translation procedure. Two bilingual translators (a dentist and a language expert) independently produced the forward translations, which were reviewed and harmonized by a panel of five experts (three dentists and two linguists) to obtain the preliminary Croatian version. Two additional translators (a dentist and a native English speaker) performed the backward translation. The expert panel compared all versions and reached consensus on the final draft. To assess clarity and comprehension, the prefinal version was tested on 20 adolescents aged 10–18 years, and minor linguistic adjustments were made based on their feedback.

A total of 254 adolescents (52% female) aged 10–18 years (median 15; interquartile range 13–16) were recruited between January and June 2022 from orthodontic clinics in Osijek, Rijeka, and Split. Each participant self‐administered the Croatian MIQ along with the PIDAQ, the JFLS, and Likert‐type scales assessing satisfaction with dental and facial appearance and perceived treatment need. The severity of malocclusion was determined using the index of orthodontic treatment need (IOTN; dental health [DHC] and esthetic components [AC]) and Little's Irregularity Index.

Statistical analyses were performed using IBM SPSS Statistics v22.0 (IBM, Armonk, NY, USA), with *p* < 0.05 considered statistically significant. Previous cross‐cultural adaptations of the MIQ have reported variability in factor structure and item performance across different populations. Therefore, exploratory factor analysis with principal component analysis and Varimax rotation was performed to examine the underlying latent structure of the Croatian version, as its structural validity had not previously been established. Internal consistency was assessed using Cronbach's *α*. Construct validity was evaluated through convergent and discriminant analyses in the cross‐sectional sample. Convergent validity was assessed using Spearman's correlations between MIQ scores and related measures, including PIDAQ, JFLS, satisfaction with dental and facial appearance, perceived treatment need, and clinical indices (IOTN and Little's Irregularity Index). Discriminant validity was evaluated using analysis of variance with the Student–Newman–Keuls post hoc test. Test–retest reliability was assessed in a separate subsample of 30 adolescents who completed the MIQ twice with a 1‐week interval and no intervening treatment; stability was evaluated using the intraclass correlation coefficient (ICC) with a 95% confidence interval. Responsiveness was analyzed using the Wilcoxon signed‐rank test to compare MIQ scores and Little's Irregularity Index before and after orthodontic treatment, and associations between changes were examined using Spearman's *ρ*. Effect size was calculated as *r* = *Z*/√*N* and interpreted as small (0.14–0.36), moderate (0.36–0.50), or large (> 0.50). Multiple linear regression analysis was used to identify factors associated with the impact of malocclusion.

No formal a priori sample size calculation was performed, as this was an observational psychometric study based on the available sample collected within a multicenter research project. The cross‐sectional validation sample (*n* = 254) exceeded commonly recommended participant‐to‐item ratios for exploratory factor analysis and questionnaire validation. Separate subsamples were used for test–retest reliability (*n* = 30) and responsiveness analyses (*n* = 36).

## 3 Results

Exploratory factor analysis of the Croatian MIQ revealed a three‐factor structure, namely psychological impact, social impact, and chewing limitation, explaining 64% of the total variance. The single‐factor solution accounted for 46% of the variance and demonstrated excellent internal consistency (Cronbach's *α* = 0.92). Deletion of the item “biting food” resulted in a slight increase in reliability (*α* = 0.93) (Table 1).

**TABLE 1:** Factor loadings of the Croatian Malocclusion Impact Questionnaire (MIQ) items after Varimax rotation.

MIQ item (description)	Component 1 psychological impact	Component 2 social impact	Component 3 chewing limitation
MIQ 4 Feeling attractive/beautiful	0.82	—	—
MIQ 3 Feeling happy	0.79	—	—
MIQ 5 Feeling self‐confident	0.78	—	—
MIQ 9 Feeling shy	0.71	—	—
MIQ 10 Smiling	0.71	—	—
MIQ 11 Laughing	0.68	—	—
MIQ 12 Seeing own photographs	0.64	—	—
MIQ 7 Feeling sad	0.63	—	—
MIQ 18 Covering teeth when smiling	0.61	—	—
MIQ 6 Feeling normal	0.57	—	—
MIQ 14 Others have nicer teeth	0.51	—	—
MIQ 8 Feeling nervous	0.45	—	—
MIQ 16 Making new friends	—	0.86	—
MIQ 17 Fitting in with friends	—	0.86	—
MIQ 15 Being bullied	—	0.84	—
MIQ 13 Speaking in public	—	0.51	—
MIQ 19 Biting food	—	—	0.78

MIQ scores correlated significantly with satisfaction with dental and facial appearance (*r* = 0.58 and 0.41; *p* < 0.001) and with self‐reported treatment need (*r* = 0.42 and 0.37; *p* < 0.001). The perceived impact of malocclusion increased proportionally with the AC of the IOTN as rated by both patients and orthodontists (*r* = 0.27 and 0.32; *p* < 0.001), while the IOTN DHC and Little's Irregularity Index showed no significant association. MIQ correlated strongly with PIDAQ domains, namely, psychological impact (*r* = 0.76), social impact (*r* = 0.74), esthetic concern (*r* = 0.64), and negatively with dental self‐confidence (*r* = −0.58; all *p* < 0.001), but not with the JFLS (Table 2).

**TABLE 2:** Correlations between MIQ total score and related psychosocial and clinical measures.

Variable	Spearman's *r*	*p*	Explanation
Satisfaction with teeth appearance	0.58	< 0.001	Positive correlation
Satisfaction with facial appearance	0.41	< 0.001	Positive correlation
Self‐reported treatment need—teeth	0.42	< 0.001	Positive correlation
Self‐reported treatment need—face	0.37	< 0.001	Positive correlation
IOTN AC (patient assessment)	0.27	< 0.001	Positive correlation
IOTN AC (orthodontist assessment)	0.32	< 0.001	Positive correlation
IOTN DHC	ns	> 0.05	No association
Little's Irregularity Index	ns	> 0.05	No association
PIDAQ—Psychological impact	0.76	< 0.001	Positive correlation
PIDAQ—Social impact	0.74	< 0.001	Positive correlation
PIDAQ—Esthetic concern	0.64	< 0.001	Positive correlation
PIDAQ—Dental self‐confidence	–0.58	< 0.001	Negative correlation
JFLS—Jaw Functional Limitation	ns	> 0.05	No association

Abbreviation: ns = nonsignificant.

Test–retest analysis in 30 adolescents showed excellent reliability (ICC = 0.93; 95% CI 0.84–0.97; *p* < 0.001). In a separate longitudinal subsample of 36 patients beginning comprehensive orthodontic therapy with fixed appliances, the MIQ score decreased by a median of 2.7 scalar points (*p* = 0.001; *r* = 0.54) after 3 months, while Little's Irregularity Index decreased by a median of 2 mm (*p* < 0.001; *r* = 0.86) (Figure 1). These changes, illustrated in Figure 1, were not influenced by sex or age. Although overall changes in MIQ and Little's Index were not correlated (*r* = 0.02; *p* = 0.908), a significant negative correlation was observed among participants with > 4 mm alignment improvement (*r* = −0.84; *p* = 0.017), indicating the questionnaire's responsiveness to clinically meaningful orthodontic improvement.

**FIGURE 1:**
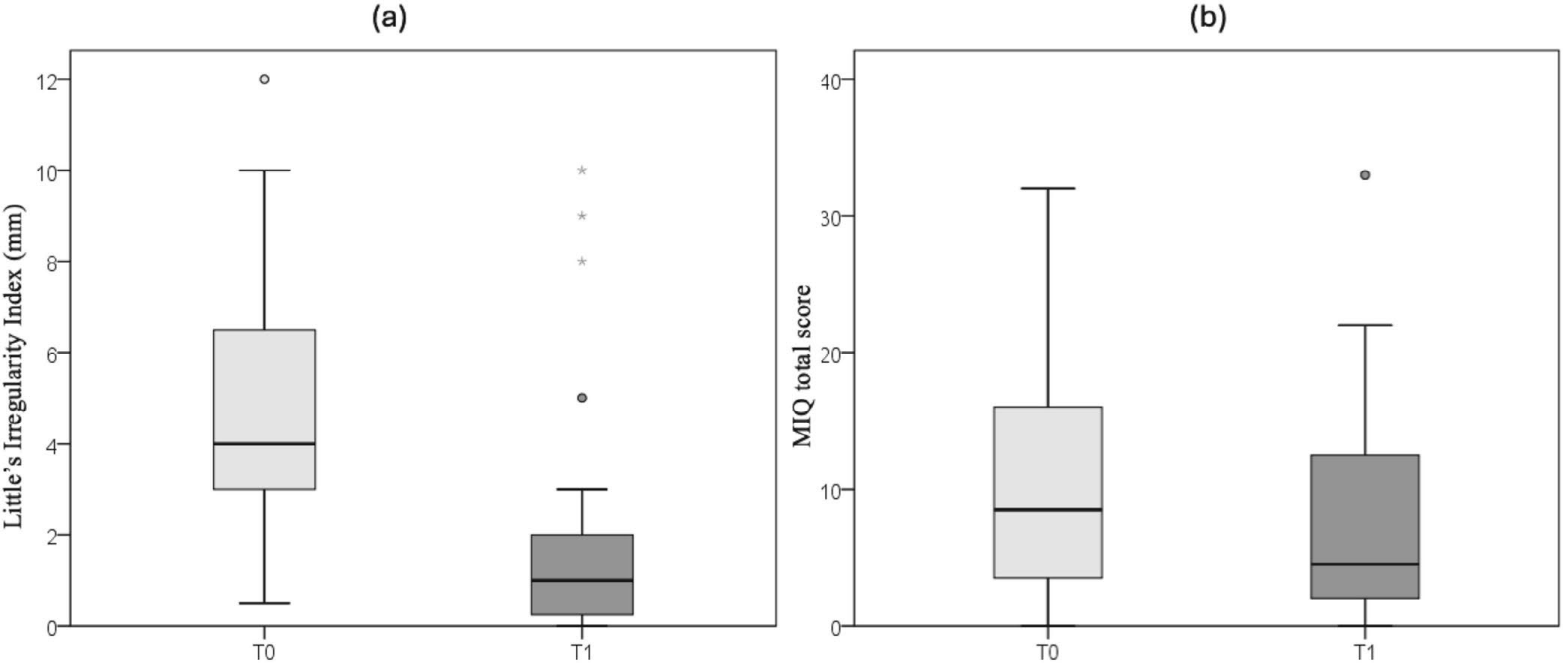
Reduction in MIQ score and incisor irregularity after 3 months of orthodontic treatment. (a) Median reduction in Little's Irregularity Index showing significant alignment improvement (*p* < 0.001). (b) Median decrease in MIQ total score showing reduced psychosocial impact (*p* = 0.001).

In multiple linear regression, predictors of malocclusion impacts were AC of orthodontic treatment need (*p* < 0.001), gender (*p* < 0.001), and age (*p* = 0.011), while controlling for the effect of DHC of orthodontic treatment need. Gender was the strongest predictor of the impact of malocclusion, explaining 13% of the variance, followed by esthetic treatment need (6%) and age (3%). Female adolescents, older participants, and those with greater self‐perceived esthetic treatment need reported higher malocclusion impacts (Table 3).

**TABLE 3:** Multiple linear regression analysis of factors associated with the impact of malocclusion.

Variable	Unstandardized coefficients		*p*	Correlations	
	*B*	Standard error		Zero‐order	Part
(Constant)	−6.6	4.1			
IOTN DHC	−0.4	0.6	0.469	0.07	−0.05
IOTN AC patient assessment	1.2	0.3	< 0.001	0.27	0.24
Gender (0 = M; 1 = F)	5.4	1.0	< 0.001	0.37	0.37
Age	0.7	0.3	0.011	0.11	0.17

*Note: R* = 0.47; *R* ^2^ = 0.22; adjusted *R* ^2^ = 0.2; *p* < 0.001.

## 4 Discussion

The present study confirmed that the perceived impact of malocclusion in adolescents is predominantly psychosocial. The impact was more pronounced in older female adolescents. Malocclusion does not appear to cause substantial functional limitations.

Although factor analysis identified three components of the MIQ, the one‐dimensional MIQ in the Croatian sample demonstrated high internal consistency, consistent with findings reported in New Zealand, China, Morocco, and Serbia [17, 18, 20, 23]. Interestingly, all these versions showed higher consistency than the original English instrument developed in the United Kingdom [16]. Therefore, the questionnaire is considered reliable as a one‐dimensional instrument. Some items partially overlapped across the three dimensions, while the item biting food stood alone in its own factor. Although its removal resulted in a marginal increase in reliability, the item was retained to preserve the functional dimension of the construct, in line with the Serbian and Finnish adaptations [17, 21]. This finding suggests that functional limitation is weakly related to malocclusion in adolescents.

Construct validity was confirmed through significant correlations with the PIDAQ, consistent with previous reports where MIQ showed the strongest association with psychosocial rather than clinical measures [16, 17, 20]. The absence of significant correlation with functional limitation (JFLS) and weak association with objective indices of malocclusion severity (IOTN DHC, Little's Irregularity Index) reflect the known discrepancy between professional assessment and self‐perceived need for orthodontic treatment [6, 7]. In present research, the impact of malocclusion is primarily psychological—mainly emotional—followed by difficulties in social interactions, heightened esthetic concerns, and reduced dental self‐confidence. As in other populations, the perceived impact of malocclusion was more closely related to esthetic concern and self‐image than to functional impairment, which was also expected due to the psychosocial nature of orthodontic motivation in adolescence [5, 16, 20]. The impact of malocclusion in our research was influenced by gender and age, with older female adolescents and those reporting a higher self‐perceived need for esthetic treatment showing greater malocclusion‐related effects. This sex‐ and age‐related pattern is consistent with previous research that showed that female adolescents report a greater psychosocial impact of dental esthetics and a higher desire for orthodontic treatment compared with males, even in the presence of comparable clinical findings [8, 9, 12]. In addition, earlier studies have indicated that girls tend to place greater importance on dental and facial appearance during adolescence, which contributes to higher self‐perceived treatment need [1, 6].

The test–retest analysis confirmed excellent temporal stability (ICC = 0.93), comparable to the Chinese, Spanish, and Finnish versions (ICC = 0.89–0.98) [19, 20, 21]. The responsiveness analysis further demonstrated that the Croatian MIQ was sensitive to improvement following orthodontic treatment, with significant reductions in both MIQ scores and dental irregularity after 3 months of orthodontic therapy with fixed appliances (Figure 1). This confirms the ability of MIQ to detect changes in quality of life resulting from orthodontic intervention, as previously suggested but rarely quantified in earlier studies [11].

This study has some limitations that are addressed. First, responsiveness of the Croatian MIQ was evaluated in a relatively small longitudinal subsample and over a short follow‐up period of 3 months; therefore, long‐term sensitivity to orthodontic treatment effects remains to be established. Second, the study population consisted of adolescents seeking orthodontic care, which limits generalizability to nontreatment‐seeking or community‐based samples. Within these limitations, the findings of this study indicate that the Croatian MIQ demonstrates good structural, convergent, and discriminant validity, as well as responsiveness to treatment‐related changes over the observed period.

## 5 Conclusion

Within the limitations of this current study, it was concluded that:

- Female gender and older age were associated with a greater impact of teeth arrangement and appearance on everyday life among adolescents.
- The Croatian version of the MIQ demonstrated good validity and reliability for assessing the psychosocial impact of malocclusion in this study.

## Author Contributions

**Matea Jurgec** and **Stjepan Špalj:** conceptualization. **Stjepan Špalj:** methodology. **Matea Jurgec, Sara Pavlović**, and **Barbara Živaljić:** validation. **Stjepan Špalj** and **Marija Čandrlić:** writing – original draft preparation. **Matea Jurgec, Sara Pavlović,** and **Barbara Živaljić:** writing – review and editing. **Stjepan Špalj:** supervision.

## Funding

Open access publishing facilitated by University of Osijek as part of the Wiley—National and University Library in Zagreb Consortium Croatian Academic and Research Libraries Consortium agreement. This study was supported by the Faculty of Dental Medicine and Health Osijek, Josip Juraj Strossmayer University of Osijek, through the institutional project “Structure of the Impact of Malocclusion on Adolescents” (FDMZ‐2021‐2022‐01).

## Ethics Statement

Ethics approval was obtained from the Ethics Committees of the Community Health Centre Osijek‐Baranja County (No. 03‐258‐2/22) and the University Hospital Centre Rijeka (Class: 003‐05/21‐1/108, No.: 2170‐29‐02/1‐21‐2).

## Conflicts of Interest

The authors declare no conflicts of interest.

## Data Availability

The data that support the findings of this study are available from the corresponding author upon reasonable request.
